# Specific Trends in Blood Utilization During the COVID-19 Pandemic: A Retrospective Analysis of a Hungarian Clinical Centre

**DOI:** 10.3390/jcm14227943

**Published:** 2025-11-09

**Authors:** Sándor Pál, Margit Solymár, Barbara Réger, Hussain Alizadeh, András Vereczkei, Tamás Kiss, Zsuzsanna Faust

**Affiliations:** 1 Department of Transfusion Medicine, Department of Laboratory Medicine, Medical School, University of Pécs, 7624 Pécs, Hungary; faust.zsuzsanna@pte.hu; 2 Department of Laboratory Medicine, Medical School, University of Pécs, 7624 Pécs, Hungary; reger.barbara@pte.hu; 3 Division of Hematology, 1st Department of Internal Medicine, Medical School, University of Pécs, 7624 Pécs, Hungary; alizadeh.hussain@pte.hu; 4 Department of Surgery, Medical School, University of Pécs, 7624 Pécs, Hungary; vereczkei.andras@pte.hu; 5 Department of Anaesthesiology and Intensive Therapy, Medical School, University of Pécs, 7624 Pécs, Hungary; kiss.tamas@pte.hu

**Keywords:** blood transfusion, COVID-19, pandemic, intensive care unit, surgery, hematology, generalized additive model, trend analysis

## Abstract

**Background/Objectives:** The COVID-19 pandemic significantly disrupted healthcare systems and blood supply chains. This study aimed to analyze blood transfusion trends across three distinct clinical departments in a Hungarian tertiary care clinical center and to examine the relationship between these trends and the pandemic waves. **Methods:** A retrospective analysis of hospitalization and transfusion data from the Departments of Anesthesiology and Intensive Therapy, Surgery, and the Division of Hematology at the University of Pécs Clinical Centre was performed between 1 January 2020, and 31 December 2023. Generalized additive models were employed to assess the association between available predictors and the odds and volume of red blood cell transfusions. **Results:** At the Department of Anesthesiology and Intensive Therapy, the median weekly ratio of transfused patients fell from 50% (pre-pandemic) to 9.76% (third wave of pandemic). COVID-19 diagnosis was associated with lower odds of receiving transfusion (OR: 0.23) and with a lower incidence rate ratio of transfused red blood cells (IRR: 0.22). At the Department of Surgery, the median weekly ratio of transfused patients was consistently low and stable (9–10%) throughout the study period. The number of patients remained relatively stable at the Division of Hematology during the study period, expressing a higher odds of receiving transfusion during the second (OR: 2.63) and fourth (OR: 1.52) pandemic waves. **Conclusions:** The pandemic’s impact on transfusion practice, driven by indirect various consequences of patient redirection and protocol modifications, was most expressed at the Department of Anesthesiology and Intensive Therapy. Similar changes in transfusion practice may be anticipated in the event of another pandemic outbreak.

## 1. Introduction

Blood transfusions are critical in managing patients in surgical, intensive care, and hematological settings. In surgical Intensive Care Units (ICUs), transfusions are common due to anemia, which increases morbidity and mortality. Research shows that transfusions are associated with lower in-hospital mortality in elderly patients and those admitted after non-cardiovascular surgeries or with severe sepsis [1]. In emergency and cardiac surgeries, transfusions are crucial for managing perioperative bleeding. While essential for life-threatening hemorrhages, they require careful evaluation [2]. Thromboelastography-guided transfusion algorithms reduce transfusion needs by identifying coagulopathy and improving hemostatic management [3,4]. For critically ill hematology patients, transfusions address anemia and tissue oxygenation needs. However, routine transfusions may not improve oxygen consumption or outcomes, necessitating personalized strategies [5]. Furthermore, they receive intensive care, including transfusions, requiring a balance between aggressive care and palliative approaches [6]. Recent advances show that standardized, multidisciplinary blood conservation programs can reduce transfusion dependence in surgeries [7]. While transfusions remain indispensable, they carry risks of infections and immune reactions.

Patient Blood Management (PBM) is a multidisciplinary approach aimed at optimizing care for patients needing blood transfusions. It is built on three pillars: optimizing hematopoiesis and managing anemia, minimizing blood loss, and optimizing physiological tolerance to anemia [8,9]. PBM strategies reduce reliance on transfusions, improving outcomes and reducing risks and costs [8,10]. During pandemics like Coronavirus Disease-19 (COVID-19), PBM becomes crucial due to challenges in blood supply. The pandemic impacts supply chains, necessitating enhanced blood management practices [11]. PBM strategies, including restrictive transfusion approaches, effectively reduce unnecessary transfusions while maintaining patient safety [12,13]. A restrictive transfusion strategy uses evidence-based guidelines to minimize blood product use [12]. These strategies conserve blood resources while improving outcomes and reducing costs [14,15]. PBM programs with monitoring systems effectively reduce transfusions and costs while enhancing care [15]. These involve managing preoperative anemia, minimizing blood loss, and following evidence-based guidelines [10]. Clinical decision support systems promote restrictive transfusion practices, demonstrating PBM effectiveness [13]. PBM is a patient-centered approach optimizing transfusion practices through evidence-based strategies. Its application during pandemics helps manage limited blood product availability effectively.

During the coronavirus pandemic, temporary changes were implemented to manage critically ill COVID-19 patients by reducing non-acute cases and restructuring care. This led to a higher primary care burden and changes in donation and transfusion policies. The COVID-19 pandemic required healthcare systems to implement changes, including postponing non-acute cases to prioritize critical COVID-19 patients [16,17]. Healthcare facilities restructured care approaches, focusing on COVID-19 patients and effective resource utilization. However, challenges included limited ICU beds and logistical barriers in obtaining tests and equipment [17]. The pandemic increased the burden on primary care services and caused stress among frontline staff [18]. This highlighted the need to support healthcare workers’ mental health during emergencies [18]. The pandemic also affected donation and transfusion policies, requiring healthcare systems to ensure blood supply safety. Emergency strategies included maintaining critical medical supplies [16]. While challenging healthcare systems globally, the pandemic provided opportunities to strengthen healthcare delivery methods [16]. The COVID-19 pandemic significantly impacted Hungary’s healthcare system during multiple waves, affecting medical care and blood transfusion services.

Blood donation and transfusion services faced significant global disruptions during the pandemic, including Hungary. Fear of virus transmission reduced blood donations, affecting both actual donations and donation intentions [19,20]. Logistical challenges like movement restrictions, closed donation sites, and prioritization of COVID-19 care impacted blood collection. Blood donation drives were restructured globally, emphasizing mobile and home-based systems [21]. Healthcare providers faced challenges in blood supply management during the pandemic. Despite reduced donations and transfusions, efforts focused on innovative models to forecast demands [22]. Hospitals adjusted operations and rescheduled procedures to optimize blood supply usage [22]. Hungary experienced similar decreases in blood donation, reflecting global patterns in regions with comparable healthcare systems. Public health messaging emphasized donation safety measures to reduce donor hesitation [23]. Countries, including Hungary, implemented measures like mobile blood drives and revised collection strategies to maintain blood services during health crises.

This retrospective study aimed to analyze transfusion trends of red blood cell concentrates across the Department of Anesthesiology and Intensive Therapy, Department of Surgery, and the Division of Hematology at the University of Pécs Clinical Center between 1 January 2020, and 31 December 2023. The secondary aim was to examine the relationship between these trends and the national pandemic waves.

## 2. Materials and Methods

### 2.1. Statistical Analysis

All data processing and statistical analyses were conducted using R statistical software (version 4.5.1) with ‘tidyverse’ package collection for data manipulation and visualisation, and ‘mgcv’ package for implementing generalized additive models (GAMs). The analysis was structured into two primary approaches: a case-based analysis focusing on individual hospitalization events and a weekly time-series analysis to examine aggregate trends.

### 2.2. Data Source and Preparation

Clinical data for the period of 1 January 2020 to 31 December 2023 were extracted from the medical documentation system of the University of Pécs, Clinical Centre, including the Department of Hematology, Department of Surgery, and the Department of Anesthesiology and Intensive Therapy. Transfusion-related information was obtained from the eTraceLine database of the Clinical Blood Transfusion Service of the Clinical Centre of the University of Pécs. The gathered information included hospitalization records (patient admission/discharge dates, demographics, ward, diagnoses) and transfusion records (blood product type, quantity, date of transfusion), based on which the analysis focused on packed red cell concentrates. National weekly COVID-19 case counts were obtained from the WHO Public Health Database [24].

Patient data were processed to create a case-level dataset, each observation representing a unique hospitalization. Key variables were obtained, including patient age at admission, sex, and assigned department. Patients were assigned to distinct pandemic periods (represented as ‘Wave’ in the dataset) based on their hospitalization dates. These periods were defined according to the official Hungarian classification, as follows: ‘Pre-pandemic’ (preceding 15 March 2020), ’First’ (15 March 2020–20 July 2020), ’Second’ (21 July 2020–21 January 2021), ’Third’ (22 January 2021–7 July 2021), ’Fourth’ (8 July 2021–25 December 2021), ’Fifth’ (26 December 2021–5 July 2022), and ’Post-pandemic’ (6 July 2022 onwards) [25].

A binary variable for COVID-19 infection status (COVID) was created for each hospitalization based on the presence of relevant ICD-10 diagnosis codes (U07.1, U07.2, B34.2). The primary outcomes for this analysis were a binary flag indicating whether a patient received a red blood cell (RBC) transfusion (Transfused_RBC_flag) and the total count of RBC units transfused per hospitalization (Total_Products_RBC).

### 2.3. Descriptive Statistics

Descriptive statistics were calculated for the case-based dataset, stratified by clinic and pandemic waves. Continuous variables, such as patient age and length of stay, were summarized using medians and interquartile ranges (IQR). Categorical variables, including sex and the percentage of patients with COVID-19, were reported as counts and percentages.

### 2.4. Case-Based Generalized Additive Models

To assess the factors associated with RBC transfusion, two sets of multivariate GAMs were fitted for each clinic, evaluating the association between multiple predictor variables and the two primary outcomes. First, a GAM with a binomial distribution and a logit link function was used to model the odds of receiving an RBC transfusion. Second, a GAM with a negative binomial distribution and a log link function was used to model the count of RBC units per hospitalization. The negative binomial distribution was chosen to account for overdispersion commonly observed when working with count data including a high frequency of zeros.

The full model formula for both outcomes was:

Outcome~Age_group + Sex + Wave + Length_of_Stay * COVID_Status

Predictor variables included age categorized into three groups (18–49—reference, 50–64, >65), sex (Female—reference, Male), the categorical Wave (Pre-pandemic—reference) variable, length of stay (in days), and the binary COVID-19 (Negative—reference) status. The interaction term Length_of_Stay * COVID_Status was included to assess whether the effect of hospitalization duration on transfusion practices differed between patients with and without a COVID-19 diagnosis. Model results were reported as odds ratios (OR) for the binomial model and incidence rate ratios (IRR) for the negative binomial model, both with 95% confidence intervals (CIs). The obtained metrics were visualized on a forest plot.

### 2.5. Model Interpretation

**Figure 1:**
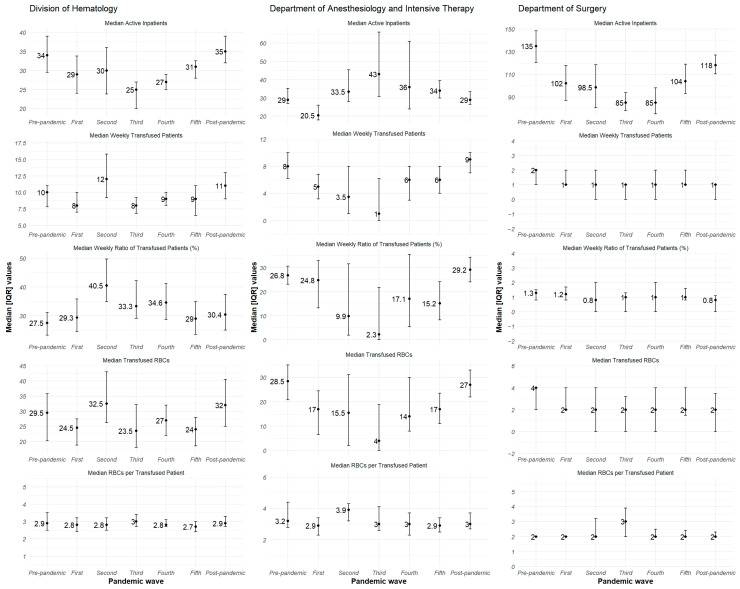
The descriptive statistics of each parameter by COVID-19 pandemic wave at the included Clinical Departments. The values are plotted as weekly data, using the median (dots annotated with their actual values) and the interquartile range (using the observed values of the Q1 and Q3 quartiles), calculated based on the weekly aggregated data of the cases included in the analysis.

The results from the GAMs are presented as OR for the binomial model and IRR for the negative binomial model. These metrics are visualized in a forest plot for a clear comparison of the predictors’ effects (Figure 1). This dual-model approach allows for a nuanced understanding, separating the factors that predict the decision to transfuse from the factors that influence the volume of transfusion once that decision is made.

The OR is used to interpret the odds of a transfusion event occurring for each predictor. It compares the odds of receiving any RBC transfusion for a given group against a reference group. An OR > 1 indicates that a patient in that group has higher odds of receiving an RBC transfusion compared to the reference group. For example, an OR of 2.0 for a specific age group means patients in that group have twice the odds of being transfused compared to the reference age group. An OR < 1 indicates lower odds of receiving a transfusion. An OR = 1 suggests no difference in the odds of transfusion compared to the reference group.

The IRR is interpreted differently. It is conditional on a transfusion event having already occurred and therefore only includes patients who received at least one unit of RBCs. This metric compares the expected count of transfused RBC units for a given group to the reference group. An IRR > 1 signifies that, among transfused patients, those in the specified group are expected to be transfused with a higher amount of RBC units compared to the reference group. For instance, an IRR of 1.5 means the rate of transfusion is 50% higher, suggesting a larger volume of blood is administered. An IRR < 1 suggests that the group received fewer units on average than the reference group. An IRR = 1 indicates no difference in the number of units transfused between the group and its reference.

### 2.6. Temporal Trend Visualization

To explore temporal patterns in transfusion practices, weekly aggregated metrics (e.g., number of active inpatients, number of patients transfused) were computed. This analysis focused on case-based modeling. Time-series data analysis required raw weekly data points to be plotted against time. To aid in the interpretation of underlying trends, a smoothed line with a 95% confidence interval were overlaid on each plot using a GAM-based smoother (geom_smooth (method = “gam”)) visual method. This was performed for descriptive visualization purposes to highlight patterns, rather than for formal statistical inference.

## 3. Results

Descriptive statistics on blood product utilization, based on weekly calculated data, revealed distinct patterns across the three clinical departments (Table 1). The Division of Hematology had the highest proportion of transfused patients, with a median weekly ratio of 32.26% and a median of 2.9 products per patient. In contrast, the Department of Surgery had a notably lower median weekly ratio of 5.1% and a median of 2.5 products per patient. The Department of Anesthesiology and Intensive Therapy occupied an intermediate position, with a median weekly transfused patient ratio of 27.2% and a median of 4.00 products per patient.

Further analysis, stratified by COVID-19 pandemic waves, indicated that these departmental patterns persisted but with temporal fluctuations (Figure 1). In the Division of Hematology, the median weekly ratio of transfused patients remained consistently high across all waves, peaking at 88.19% during the third wave before a subsequent decline. The highest median weekly ratio of transfused patients could be observed during the second wave of the pandemic (41.0%). For the Department of Anesthesiology and Intensive Therapy, the median weekly transfused patient ratio was highest in the post-pandemic period (32.1%), which was close to the values observed in the pre-pandemic period and during the first wave. This unit experienced a substantial decrease to 4.0% during the third wave, and then showed a moderate increase in later periods. The Department of Surgery maintained a relatively stable and low median ratio of transfused patients throughout all pandemic waves, with minimal variation. These findings suggest that the COVID-19 pandemic differentially influenced blood transfusion practices depending on the specific clinical context and patient population.

### 3.1. GAM Results by Clinic

**Figure 2:**
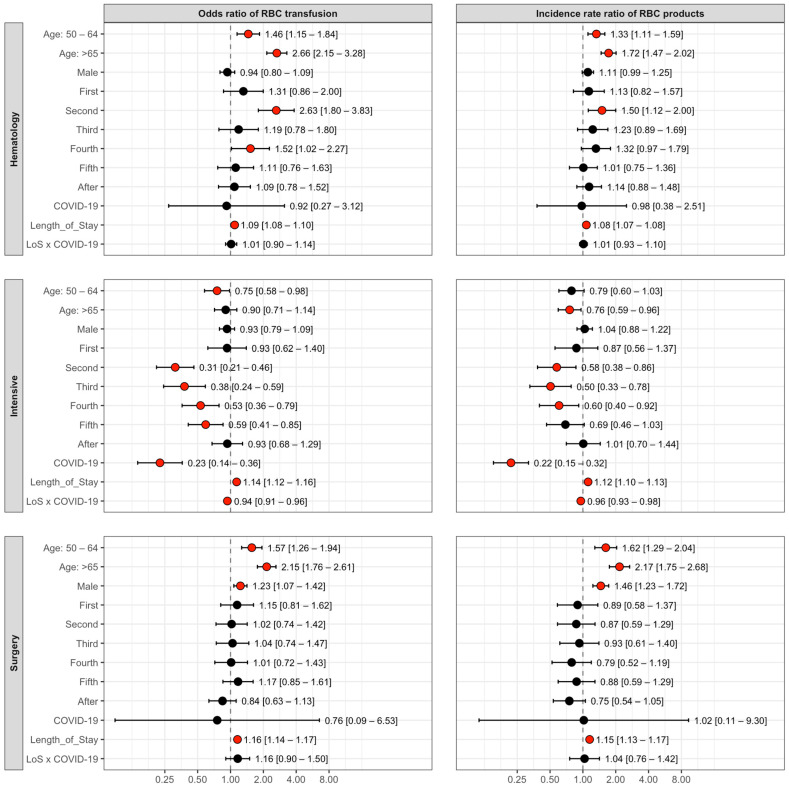
The odds ratios (OR) and incidence rate ratios (IRR) of the selected predictors. The left side of the plot displays the odds ratios, which address the likelihood of receiving a transfusion (based on a binary-yes/no-event). The baseline odds are by default at 1, representing the reference group for each predictor. The right side of the plot shows the incidence rate ratios, which concern the quantity of blood received among only those patients who were transfused. This part of the analysis answers the following question: “If a patient receives a transfusion, what is their chance of getting a higher volume of blood compared to the control group?”. Significant IRR/OR values are marked with red coloured dots.

Figure 2 represents the forest plots of OR with 95% CI of the predictors by clinic for the odds of RBC transfusion and odds of increased number of transfused RBCs.

At the Division of Hematology, patient age was a significant predictor of transfusion. Compared to patients aged 18–49, the odds of receiving a transfusion were 1.46 times higher for patients aged 50–64 and 2.66 times higher for those over 65. A similar trend was observed for the volume of RBCs among transfused patients, the rate of units being significantly higher in older age groups. The odds of receiving a transfusion were significantly higher during the second (OR = 2.63, 95% CI: 1.80–3.83) and fourth (OR = 1.52, 95% CI: 1.02–2.27) waves compared to the pre-pandemic period. For patients who received blood, the rate of transfused units was 1.50 times higher (a 50% increase) during the second wave. Each additional day of hospitalization increased the odds of transfusion by 9% (OR = 1.09, 95% CI: 1.08–1.10) and the incidence rate ratio of RBCs by 8% (IRR = 1.08, 95% CI: 1.07–1.08). Patient sex and COVID-19 status were not associated with a significant difference in either the odds or rate of transfusion.

In the Department of Anesthesiology and Intensive Therapy, patients aged 50–64 had 25% lower odds of receiving a transfusion (OR = 0.75, 0.58–0.98) compared to the 18–49 age group. Among those transfused, patients over 65 received on average 24% fewer RBCs (IRR = 0.76, 95% CI: 0.59–0.96). Compared to the pre-pandemic period, the odds of transfusion were significantly lower during the second through fifth waves, with the largest reductions in the second (69% lower odds; OR = 0.31, 95% CI: 0.21–0.46) and third (62% lower odds; OR = 0.38, 95% CI: 0.24–0.59) waves. A longer hospital stay was a strong predictor, increasing the daily odds of transfusion by 14% (OR = 1.14, 95% CI: 1.12–1.16) and the daily IRR of RBC units by 12% (IRR = 1.12, 95% CI: 1.10–1.13). In contrast, a COVID-19 diagnosis was associated with 77% lower odds of transfusion (OR = 0.23, 95% CI: 0.14–0.36) and a 78% lower IRR of RBCs (IRR = 0.22, 95% CI: 0.15–0.32) compared to non-COVID-19 patients. A significant interaction was also found. In the case of the patients suffering from COVID-19, the positive effect of a longer stay on both the odds of transfusion and IRR of transfusion was significantly diminished, resulting in slightly but significantly lower OR of transfusion and IRR of RBCs.

At the Department of Surgery, older age groups and male patients had higher odds of receiving RBC transfusions and were administered a higher rate of units compared to their respective reference groups (18–49 years and female). The pandemic waves did not significantly alter transfusion practices in this department compared to the pre-pandemic baseline. Length of stay was a highly significant predictor, where each additional day increased the odds of transfusion by 16% (OR = 1.16, 95% CI: 1.14–1.17) and the rate of units by 15% (IRR = 1.15, 95% CI: 1.13–1.17). A COVID-19 diagnosis did not have a statistically significant impact on the odds or rate of RBC transfusion for surgical patients.

### 3.2. Visual Trend Analysis of Weekly Active Inpatients, Transfused Patients, and the Ratio of Patients Transfused

**Figure 3:**
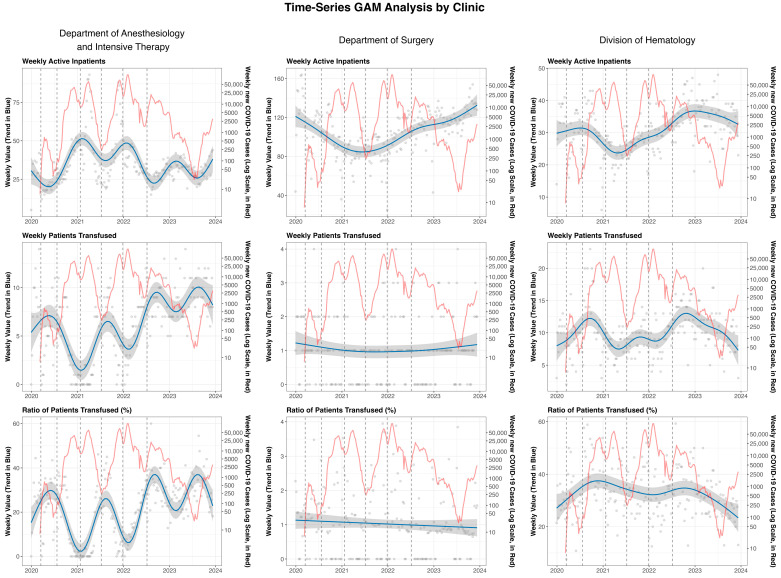
Smoothed trendlines (blue) with 95% CI (grey ribbon) of weekly active inpatients (count data), weekly patients transfused (count data), and ratio of patients transfused (percentage) shown by facets for each included department. Grey dots represent the weekly count or percentage data, on which the smoothed trendlines were produced. The weekly logarithmic representation (red line) of novel COVID-19 cases in Hungary was also plotted to aid interpretation.

Figure 3 depicts the smoothed trendline of the selected outcomes faceted by each analyzed clinical department.

The Department of Anesthesiology and Intensive Therapy demonstrated a consistent positive trend between new COVID-19 case numbers and clinical activity. The association was observable across all waves, confirming that as community COVID-19 cases increased, the weekly active inpatients in the ICU also rose. We found an inverse trend between the number of new COVID-19 cases per week and the number of patients transfused per week. The number of weekly transfused patients was so low (2–4 patients per week) that the ratio of patients transfused showed a similar negative trend with the new COVID-19 cases.

The number of patients in the Department of Surgery decreased during all COVID-19 waves. As new COVID-19 cases rose, the number of active inpatients in the surgery department decreased, revealing an inverse trend between them. In the post-COVID-19 period, the number of weekly active inpatients increased, even exceeding pre-COVID-19 values by the end of the analyzed interval. The weekly patients transfused and the rate of transfusion remained low, independent of the pandemic’s waves.

In the Division of Hematology, the weekly number of active inpatients decreased during the second, third, and fourth COVID-19 waves, and by the post-COVID era, a continuous rising trend could be observed. During the third, fourth, and fifth waves, the median number of patients transfused weekly was generally comparable to that of the pre-pandemic period. However, the rate of patients receiving transfusions increased in parallel with the rise in new COVID-19 case numbers.

## 4. Discussion

A central finding of this study is that the impact of the COVID-19 pandemic on transfusion practice was not uniform but rather highly specific to the clinical context of each analyzed department. The three clinical departments illustrated how systemic pressure was mediated through different clinical contexts. The COVID-19 pandemic profoundly transformed healthcare in hospitals, and its impact on transfusions was statistically significant and multilayered [25,26,27,28,29,30]. The observed changes were predominantly the indirect consequences of a healthcare system undergoing temporary radical reorganization, which included the redirection of patient flows and modifications in clinical protocols in response to the crisis.

The Department of Anesthesiology and Intensive Therapy presented a complex scenario. The peak inpatient census during the third wave coincided with a significant reduction in transfusion activity. This was driven by a fundamental shift in the ICU’s patient case-mix [31]. The unit was predominantly occupied by patients with severe COVID-19, who, despite their critical condition, required significantly fewer red blood cell (RBC) transfusions compared to the typical ICU population of major trauma, complex post-surgical, or septic shock patients [32,33,34]. Research investigating transfusion practices in the management of COVID-19 patients has documented a transfusion frequency rate of 10% to 11% [35,36]. The GAM analyses of this study strongly support these findings, revealing that COVID-19-positive patients in the ICU were significantly less likely to receive RBCs (OR 0.21) or increased transfusion volumes (OR 0.19). This occurred even though the COVID-19 ICU population had a higher mean age. The systemic response—prioritizing ICU beds for pandemic-related cases—effectively displaced the patient population that traditionally drives transfusion demand. During the post-pandemic period this trend reversed and began to normalize [37,38,39].

The Department of Surgery provides a clear example of how the redirection of patient care impacted resource utilization. A significant reduction in active inpatients was observed during the peak waves of the pandemic, which directly correlated with the well-documented postponement of elective surgeries to conserve resources and minimize viral transmission [40,41]. Although the absolute number of transfusions decreased in line with the reduced patient load, the proportion of patients receiving transfusions remained remarkably stable. The Generalized Additive Model (GAM) results corroborate this, showing a significant decline in inpatient numbers as community COVID-19 cases rose, yet no statistically significant change in transfusion rates. This suggests that for the non-elective, urgent surgical cases that continued, standard transfusion practices were maintained. The observed impact was therefore attributable almost entirely to a reduction in surgical volume rather than a change in clinical decision-making for transfusions. Furthermore, our analysis showed that in the surgical cohort, older age was associated with higher odds of receiving RBC transfusions, a finding consistent with existing data on the increased risk of anemia and perioperative transfusion needs in these demographics. The overrepresentation of acute, severe cases during this period likely amplified this effect [42,43]. The observation of increased odds of transfusion among males may be attributed to other transfusion-related risks and acute interventions that were not accounted for in this study.

In the Division of Hematology, where patients frequently have non-deferrable transfusion requirements, the pandemic’s influence manifested differently. Although inpatient numbers remained relatively stable, the GAM analysis indicated that rising community COVID-19 cases were associated with shifts in transfusion practices. During the second and fourth waves, the odds of RBC transfusion increased, and the second wave saw a significantly higher amount of RBCs transfused per patient. The higher OR and IRR of RBC transfusion among patients with hematological diseases reflects an increased blood demand among these patients, possibly because during these periods a higher rate of more severe patients were treated at this unit. These systemic changes were also reported by Velázquez-Kennedy et al. [27] and Riley et al. [44]. The reduced transfusion activities observed at the Department of Surgery and at the Department of Anesthesiology and Intensive Therapy were possibly in correlation with the weekly number of hospitalizations and different patient routes during pandemic waves, transfusion activity at the Division of Hematology remained high due to nonadjustable transfusion among the hematological patients [34]. Faced with potential shortages, clinicians may have adopted more cautious transfusion thresholds, reserving products for the most critical needs. The higher odds of transfusion observed among older hematology patients are consistent with the known correlation between advanced age, comorbidities, and anemia [1,27,43]. Importantly, and in contrast to the ICU, a positive SARS-CoV-2 test among hematology patients did not independently alter their odds of transfusion, reinforcing that the impact was systemic rather than a direct result of the virus’s pathophysiology in this specific cohort.

This study is subject to limitations. As a single-center retrospective analysis, the findings may not be generalizable to other healthcare institutions with different patient populations or operational responses to the pandemic. The reliance on administrative data, including ICD-10 codes for defining COVID-19 status, may be prone to coding inaccuracies and lacks detailed clinical information such as pre-transfusion hemoglobin levels, specific transfusion triggers, or patient severity scores. The binary classification of COVID-19 infection status (positive/negative) does not capture important cofactors such as repeated infections. The vaccination status of the patients, which may have been influencing the clinical presentation of these cases, could not be gathered. Consequently, it was not possible to fully account for all clinical variables that may have influenced transfusion decisions, and the potential for unmeasured confounding variables is present. Thus, the possible influence of these predictors on the clinical outcomes could not be analyzed. These limitations underscore the need for cautious interpretation of the results.

The strengths of our study are the wide time frame, allowing the analysis of the full pandemic period in Hungary, the high case numbers included, the two different statistical approaches, and the inclusion of data from three different departments of a tertiary care hospital.

Measures of PBM in Hungary, including the Clinical Center of the University of Pécs, were continuously adopted, with scientific publications on PBM being published in national Hungarian scientific journals since 2018 [45,46,47,48,49,50,51]. During the COVID-19 pandemic, formal or official recommendations did not encompass the modification of transfusion thresholds.

During the pandemic, many studies focused on the analysis of the blood supply changes, and on the overall balance between blood product supply and demand rather than focusing on the blood product usage in specific clinical units [52,53]. The available studies emphasized the challenges of blood collection imposed by the pandemic. According to the results of this study, rising COVID-19 cases were a statistically significant predictor of transfusion metrics across the hospital. This correlation does not imply a direct causal burden on blood banks from the infected patients themselves (outside of the ICU context). Instead, the pandemic exerted its influence indirectly. In the Department of Surgery, the effect was a near-total shutdown of elective activity. In the ICU, it was the replacement of a higher blood transfusion demand population with a lower one. Among hematological cases, it prompted a strategic adaptation of clinical practice in response to perceived systemic risk. Thus, the significant impact on transfusion medicine was less about managing an overwhelming demand for blood products and more about adapting to the profound operational and strategic constraints imposed by a global health crisis.

## 5. Conclusions

The impact of the COVID-19 pandemic on transfusion practice was significant and highly specific to each clinical department, with the most pronounced effects observed in the Department of Anesthesiology and Intensive Therapy. Both the use of red blood cell concentrates and the number of patients transfused were significantly reduced during the COVID-19 pandemic at this department. Similar changes in transfusion practice may be anticipated in the event of another pandemic outbreak.

## Figures and Tables

**Table 1:** Descriptives of the selected parameters by departments during the study period. The values are reported as weekly data in the following format: median [Q1–Q3].

	Active Inpatients ^1^	Transfused Patients ^1^	Transfused Blood Product ^1^	Product Per Transfused Patient ^3^	Ratio of Transfused Patients ^2^
Division of Hematology	31.0 [26.0–36.0]	29.0 [23.0–37.0]	10.0 [8.0–12.0]	2.9 [2.6–3.2]	32.2 [26.6–40.0]
Department of Anesthesiology and Intensive Therapy	31.0 [27.0–39.0]	25.0 [13.0–34.0]	8.0 [4.0–10.0]	3.0 [2.5–3.7]	27.2 [13.0–35.6]
Department of Surgery	105.0 [88.0–121.0]	14.0 [9.0–18.0]	5.0 [4.0–7.0]	2.5 [2.0–2.8]	5.1 [3.8–6.7]

^1^—count; ^2^—rate; ^3^—percentage.

## Data Availability

The data that support the findings of this study are not publicly available due to restrictions imposed by the University of Pécs. However, data are available from the corresponding author upon reasonable request and with the permission of the University of Pécs.

## Abbreviations

The following abbreviations are used in this manuscript:

CI	Confidence Interval
COVID-19	Coronavirus Disease 2019
GAM	Generalized additive model
ICD-10	International Classification of Diseases, Tenth Revision
ICU	Intensive care unit
IQR	Interquartile range
IRR	Incidence rate ratio
OR	Odds ratio
PBM	Patient Blood Management
RBC	Red blood cell
WHO	World Health Organization
